# Encapsulation of Bee Pollen Phenolics with β-Cyclodextrin: Effects on Antioxidant Activity, Antimicrobial Properties, and Digestive Stability

**DOI:** 10.3390/foods15061047

**Published:** 2026-03-16

**Authors:** Aslı Akdas, Deniz Günal-Köroğlu, Dilara Devecioglu, Esra Capanoglu, Funda Karbancioglu-Guler, Gulay Ozkan

**Affiliations:** 1 Department of Food Engineering, Faculty of Chemical and Metallurgical Engineering, İstanbul Technical University, 34469 Istanbul, Türkiyedevecioglud@itu.edu.tr (D.D.); ozkangula@itu.edu.tr (G.O.); 2 Department of Food Engineering, Faculty of Engineering, Sakarya University, 54050 Sakarya, Türkiye; denizkoroglu@sakarya.edu.tr

**Keywords:** phenolic extract, phenolic compounds, antioxidant activity, in vitro gastrointestinal digestion, bioaccessibility

## Abstract

Bee pollen is a natural product with multifunctional properties, containing abundant bioactive compounds, especially phenolic acids and flavonoids, which are largely responsible for its antioxidant and antimicrobial activities. In this study, the bioactive composition, antioxidant capacity, encapsulation efficiency, antimicrobial activity, and gastrointestinal stability of bee pollen extract (PE) were investigated. The pollen extract exhibited high total phenolic (2817 mg GAE/100 g) and flavonoid contents (5255 mg QE/100 g), along with strong antioxidant activity (DPPH: 4305 mg TE/100 g; CUPRAC: 3685 mg TE/100 g). To improve the stability and bioaccessibility of phenolic compounds, PE was encapsulated using β-cyclodextrin (BCD) at different weight ratios. Among the formulations, the PE:BCD ratio of 1:2 showed the highest encapsulation efficiency (64%) and favorable physicochemical properties, including higher particle size and more negative zeta potential values, indicating good colloidal stability. Antimicrobial activity was evaluated for PE, BCD-only, and the selected PE-loaded formulation (1:2, *w*:*w*). Encapsulation led to a modest reduction in antimicrobial activity compared to free PE (6.25–50 mg/mL); however, the encapsulated formulation still exhibited considerable antibacterial effects against both Gram-positive and Gram-negative strains (25–50 mg/mL). Furthermore, in vitro gastrointestinal digestion indicated that BCD encapsulation substantially enhanced the bioaccessibility of total phenolics (81%) and antioxidant capacity (DPPH: 48%; CUPRAC: 76%), particularly during the intestinal stage. Phenolic profiling showed that chlorogenic acid and quercetin derivatives remained relatively stable throughout digestion. Overall, encapsulation with BCD effectively safeguarded pollen phenolics, improved their gastrointestinal stability, and increased bioaccessibility, highlighting the potential of encapsulated bee pollen as a functional food ingredient or nutraceutical.

## 1. Introduction

Bees are highly organized social insects belonging to the Apidae family and play a crucial role not only in pollination but also in the production of nutritionally and pharmacologically valuable natural products [1]. Bee-derived products, including honey, propolis, royal jelly, and pollen, have been used since ancient times for both dietary and therapeutic purposes. In recent years, increasing attention has been directed toward bee products as natural sources of bioactive compounds with antioxidant potential, capable of counteracting oxidative stress, a key contributor to the development of chronic and degenerative diseases [2].

Bee pollen is formed through the aggregation of pollen grains collected from various botanical sources and mixed with nectar and salivary enzymes secreted by honeybees [3]. Owing to its complex composition, bee pollen contains a wide range of primary and secondary metabolites, including proteins, amino acids, lipids, carbohydrates, vitamins, minerals, and polyphenolic compounds [4]. These constituents are responsible for its diverse biological activities, such as antioxidant, anti-inflammatory, antimicrobial, anticancer, hepatoprotective, and immunomodulatory effects [3].

Bee pollen is recognized as a rich source of phenolic acids and flavonoids with high antioxidant potential. The antimicrobial activity of pollen-derived phenolics is associated with multiple complementary mechanisms. Phenolic acids and flavonoids can disrupt microbial cell membranes, increasing permeability and causing leakage of intracellular constituents, ultimately leading to cell death [5,6]. Many phenolics also promote the intracellular generation of reactive oxygen species (ROS), which damage DNA, proteins, and membrane lipids [5]. In addition, certain phenolic acids contribute to extracellular pH reduction, thereby creating unfavorable conditions for microbial growth [7]. Enzyme inhibition represents another key mechanism, as phenolics can interfere with enzymes involved in energy metabolism and cell wall synthesis [6]. Some compounds are further reported to interact directly with microbial DNA, impairing replication and transcription processes [5]. Collectively, these mechanisms explain the broad-spectrum antibacterial potential of bee pollen phenolics beyond their well-known antioxidant activity.

Despite its high bioactive potential, the application of bee pollen in food and nutraceutical systems is limited by the poor stability and bioavailability of its phenolic compounds. Factors such as low water solubility, susceptibility to gastrointestinal degradation, and limited intestinal absorption restrict the effective utilization of pollen polyphenols [8]. Furthermore, structural modification or degradation of phenolics under gastrointestinal conditions may reduce not only their antioxidant capacity but also their antimicrobial potency. Limited antimicrobial efficacy under gastrointestinal conditions can be attributed to several factors. Phenolic compounds may undergo structural degradation or transformation due to pH variations and enzymatic activity in the gastrointestinal tract, leading to reduced biological activity. In addition, microbial metabolism in the intestinal environment can convert phenolics into smaller metabolites with lower antimicrobial potency [9]. Simulated gastrointestinal digestion may promote structural modifications, oxidation, or binding of phenolic compounds to proteins, lipids, and digestive enzymes, reducing their availability to penetrate microbial cell walls or reach intracellular targets [10]. Consequently, despite showing inhibitory activity before digestion, the extracts lose their antimicrobial effectiveness after in vitro digestion due to the decreased concentration of free and bioactive phenolics [11] These processes help explain the loss of inhibitory activity observed after in vitro digestion.

Encapsulation has emerged as an effective strategy to overcome these limitations by protecting sensitive bioactives, enhancing their stability, and enabling controlled release [12]. Among encapsulation materials, β-cyclodextrins (BCDs) are widely used due to their ability to form inclusion complexes with phenolic compounds, thereby improving the solubility and physicochemical stability of phenolic compounds while protecting them against environmental and gastrointestinal degradation [13]. Moreover, BCDs enable controlled release, which may enhance bioaccessibility and preserve antimicrobial efficacy at the target site [14,15]. However, the encapsulation material itself exhibits no intrinsic biological activity; for example, it does not display antioxidant properties [16]. Therefore, incorporating microbial stability aspects and protective delivery systems is essential when discussing the limited antimicrobial effectiveness of phenolic-rich extracts following gastrointestinal digestion.

In this context, encapsulation of bee pollen not only facilitates its incorporation into functional food systems but also enhances its antimicrobial and surface-active properties, broadening its potential applications in food processing and preservation [17,18]. Therefore, investigating the encapsulation of bee pollen phenolics and evaluating their bioaccessibility and antimicrobial efficacy is of considerable scientific and industrial interest.

In the present study, bee pollen extract obtained from the Eastern Anatolia region of Türkiye was encapsulated with different ratios of BCD. This study was designed to evaluate the effects of encapsulation on physicochemical properties, phenolic profile, antioxidant capacity, antimicrobial activity and bioaccessibility after in vitro gastrointestinal digestion. BCD encapsulation was expected to enhance the stability and bioaccessibility of phenolic compounds and, consequently, to preserve or potentially improve antimicrobial activity by protecting bioactive compounds against gastrointestinal degradation.

## 2. Materials & Methods

### 2.1. Materials

Bee pollen was obtained from the Eastern Anatolia region of Türkiye. BCD (Sigma-C4767; ≥97%; CAS No: 7585-39-9), Folin–Ciocalteu reagent, (+)-catechin, ethanol (96%), sodium carbonate (Na_2_CO_3_), Mueller–Hinton Broth (MHB, Merck No.: 1.10293), Mueller–Hinton Agar plates (MHA, Merck No.: 1.03872), and other analytical-grade chemicals were purchased from Merck (Darmstadt, Germany). Pepsin (541 U/mg, EC 3.4.23.1), pancreatin (8× USP, EC 232.468.9), bile extract porcine (B8631), gallic acid monohydrate (398225, 98%), 1,1-diphenyl-2-picrylhydrazyl (DPPH), 6-hydroxy-2,5,7,8-tetramethylchroman-2-carboxylic acid (Trolox), resazurin (R7017), and 2,9-dimethyl-1,10-phenanthroline (neocuproine, ≥98%, N1501) were obtained from Sigma-Aldrich (St. Louis, MO, USA). For antimicrobial analyses, the bacterial strains *Bacillus cereus* ATCC 11778, *Escherichia coli* ATCC 25922, *Staphylococcus aureus* ATCC 25923, and *Salmonella* Typhimurium ATCC 0402 were used.

### 2.2. Preparation of Powdered Pollen Phenolic Extracts (PE)

Phenolic compounds were extracted from pollen following the procedure reported by Yeşiltaş et al. [19]. Pollen samples were ground for 1 min using a coffee grinder (Sinbo, SCM 2934, Istanbul, Türkiye). Subsequently, 2 g of the powdered material was combined with 15 mL of methanol and kept under dark conditions at ambient temperature for three days to allow for solvent extraction. During this period, the mixtures were vortexed once per day and additionally treated in an ultrasonic water bath for 15 min to improve the extraction yield. At the end of the extraction period, the samples were subjected to centrifugation (2700 g, 10 min, 4 °C). The resulting supernatant was carefully separated and maintained at 4 °C until further analyses were performed. The bioactive characteristics of the PE were evaluated as described in Section 2.6. “Determination and Identification of Phenolic Compounds”.

### 2.3. Preparation of Inclusion Complexes

Based on the protocol described by dos Santos Ferreira et al. [20], inclusion complexes were produced by co-precipitation followed by freeze-drying. Briefly, BCD was dispersed in 50 mL distilled water and maintained under magnetic stirring at 50 °C until complete dissolution. Although moderate temperatures such as 50 °C are widely applied to facilitate encapsulation complex formation, extended exposure to heat may influence the stability of some thermolabile phenolic compounds. Subsequently, PE was added to the BCD solution in amounts corresponding to PE: BCD weight ratios of 1:1, 1:2, and 1:3. After the addition of PE, the mixture was stirred at 50 °C for 3 h and then continuously stirred at room temperature (25 °C) for 24 h.

### 2.4. Characterization of Inclusion Complex

#### 2.4.1. Zeta Potential and Particle Size Distribution

The particle size characteristics and zeta potential of the inclusion complexes were analyzed following the procedure reported by Catchpole et al. [21]. Particle size distribution was determined using a static light scattering system (Mastersizer 2000, Malvern Instruments, Malvern, UK), whereas the zeta potential was measured with a zeta potential analyzer (Zetasizer 2000, Malvern Instruments). Before the measurements, PE:BCD complex solutions and BCD solutions were prepared in distilled water at a concentration of 0.1%. All measurements were performed in triplicate.

#### 2.4.2. Encapsulation Efficiency

To determine encapsulation efficiency, 50 mg of the inclusion complexes was dissolved in 10 mL of 80% ethanol and filtered through a 0.45 μm membrane. Total phenolic content of filtrate was determined as described in “Bioactive Properties” and encapsulation efficiency (%) was calculated using the following equation:

$$Encapsulation\;Efficiency\left(\%\right)=\frac{Phenolic\;content\;in\;the\;inclusion\;complex}{Phenolic\;content\;in\;PE}\times 100$$

It should be noted that calculating encapsulation efficiency based solely on total phenolic content may not fully reflect the retention of specific antimicrobial-active phenolic compounds, as different phenolics can vary in their encapsulation behavior and thermal or solvent stability. Therefore, while total phenolics provide a general measure of encapsulation success, the actual antimicrobial efficacy may depend on the retention of key bioactive constituents. Following characterization, the inclusion complexes with the optimal active ingredient ratio (PE:BCD) were selected for subsequent in vitro gastrointestinal digestion and antimicrobial analyses.

#### 2.4.3. Determination of Antimicrobial Activity

The antimicrobial activity of PE, BCD-only, and the selected PE-BCD inclusion complex formulation (PE:BCD, 1:2, *w*:*w*) was tested against two Gram-positive bacteria, *Staphylococcus aureus* ATCC 25,923 and *Bacillus cereus* ATCC 11778, and two Gram-negative bacteria, *Escherichia coli* ATCC 25,922 and *Salmonella* Typhimurium ATCC 0402. All bacterial cultures were inoculated in Mueller–Hinton Broth (MHB, Merck No.: 1.10293, Darmstadt, Germany) at 37 °C for 24 h (N-Biotek, Bucheon, Republic of Korea). The resulting suspensions were adjusted to approximately 1 × 10^5^ CFU/mL using sterile 0.85% NaCl solution (*w*/*v*).

The Minimum Inhibitory Concentration (MIC) and Minimum Bactericidal Concentration (MBC) values were determined by the broth microdilution method using 96-well microplates [22]. Test samples were prepared in MHB by two-fold serial dilution over a concentration range of 50–0.098 mg/mL. Each well contained 180 µL of the diluted sample and 20 µL of bacterial suspension. Optical density at 600 nm (OD_600_) was measured with a microplate reader (BioTek Technologies, Winooski, VT, USA) before and after incubation at 37 °C for 18–24 h. Wells with MHB and bacteria alone served as the positive control, while wells containing a sample but no bacteria served as negative controls. The MIC was defined as the lowest sample concentration at which no visible microbial growth occurred.

For MBC determination, 20–30 µL aliquots from wells without visible growth were plated onto MHA and incubated at 37 °C for 18–24 h. The MBC corresponded to the lowest concentration at which no bacterial colonies formed, indicating complete bactericidal activity [22].

To further verify MIC and MBC, a resazurin assay, which indicates the metabolic activity of viable microorganisms, was performed. After sampling for MBC, 10 µL of resazurin solution (1 mg/mL) was added to each well and incubated at 37 °C for 3–4 h. A color change from blue to pink indicated bacterial growth, whereas the persistence of blue color indicated inhibition of metabolic activity [23]. Accordingly, the MIC was confirmed by the absence of a color change from blue to pink, and for MBC determination, aliquots from wells that remained blue were subcultured onto antimicrobial-free agar to confirm bactericidal activity.

### 2.5. In Vitro Gastrointestinal Digestion

**Figure 1:**
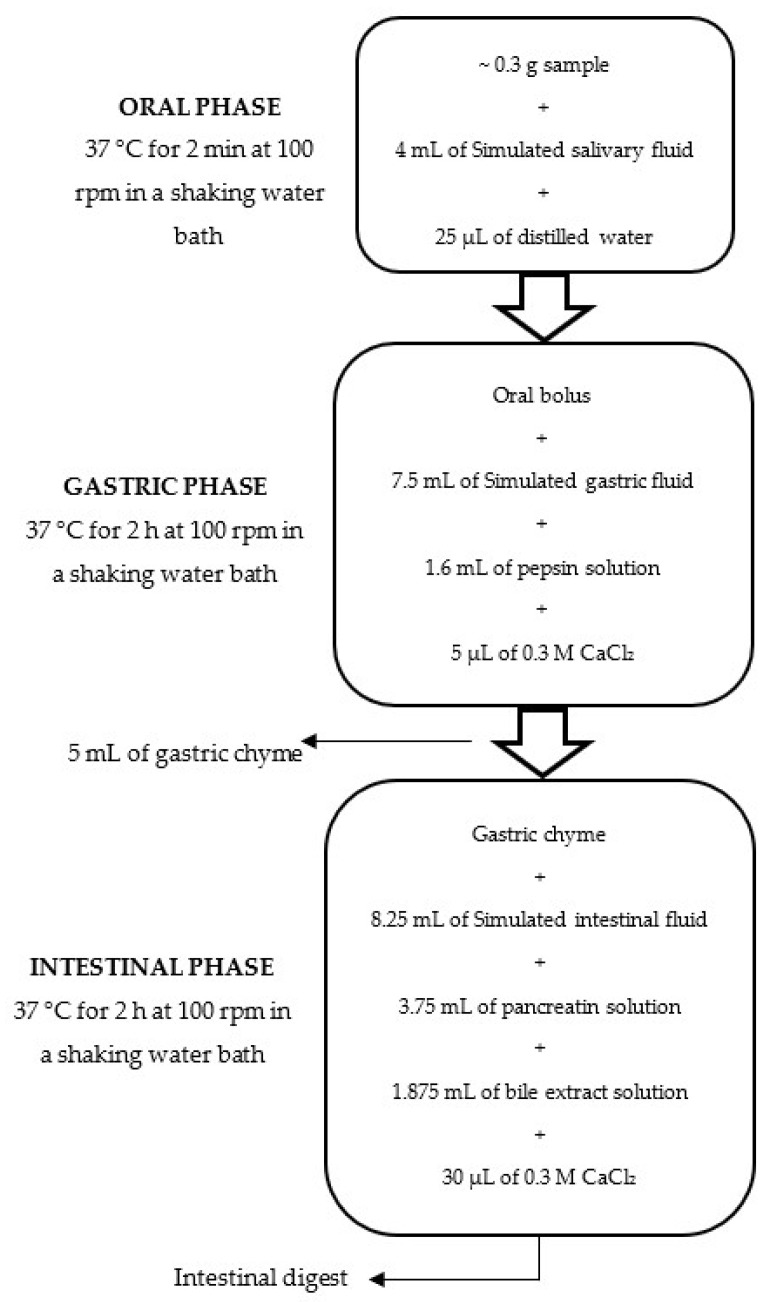
Schematic representation of in vitro gastrointestinal digestion.

Simulated gastrointestinal digestion was performed according to the standardized INFOGEST protocol reported by Minekus et al. [24], with minor modifications. All in vitro gastrointestinal digestion experiments were conducted under microbiologically controlled conditions, using sterile equipment and solutions, to prevent unintended microbial growth during the simulation. Simulated digestive fluids and enzyme solutions were prepared strictly in accordance with the original Minekus method, and all solutions were pre-equilibrated to 37 °C prior to use. This method sequentially simulates oral, gastric, and intestinal digestion phases. A schematic overview of the methodology is presented in Figure 1.

To correct for any potential interference originating from the simulated digestive fluids, a blank sample (prepared using distilled water instead of sample material) was incubated under identical conditions. All collected digesta were centrifuged at 23,000× *g* for 30 min at 4 °C (Hettich, Tuttlingen, Germany). The supernatants were collected and stored at −20 °C until further analysis.

### 2.6. Determination and Identification of Phenolic Compounds

The phenolic profile of the encapsulated samples was analyzed by HPLC according to the method described by Capanoglu et al. [25]. Prior to injection, samples were filtered through 0.45 μm membrane filters and injected into a Waters 2695 HPLC system (Waters, Milford, MA, USA) equipped with a photodiode array (PDA) detector (Waters 2996). Chromatographic separation was carried out on a Supelcosil LC-18 reversed-phase column (25 cm × 4.60 mm, 5 μm particle size; Sigma-Aldrich, St. Louis, MO, USA). The mobile phase consisted of trifluoroacetic acid (TFA, 1 mL/L) in Milli-Q water (eluent A) and TFA (1 mL/L) in acetonitrile (eluent B). Analyses were performed at a flow rate of 1 mL/min with an injection volume of 10 μL, and spectral data were recorded at 280, 312, and 360 nm. A linear gradient elution program was applied as follows: 95% solvent A and 5% solvent B at 0 min; 65% solvent A and 35% solvent B at 45 min; 25% solvent A and 75% solvent B at 47 min; and a return to initial conditions at 54 min.

Quantification of phenolic acids was performed using their respective authentic standards. Identification and quantification of polyphenols were achieved by comparing the retention times, UV spectra, and peak areas of sample extracts with those of reference standards, including *t*-cinnamic acid, *p*-coumaric acid, caffeic acid phenethyl ester (CAPE), quercetin-3-O-glucoside and quercetin. All analyses were performed in triplicate, and the results were expressed as mg per 100 g of dry weight (mg/100 g dw).

### 2.7. Bioactive Properties

#### 2.7.1. Total Phenolic Content (TPC)

TPC of the samples was determined using the Folin–Ciocalteu method [26], and applied as reported in reference [27]. A calibration curve of gallic acid was used to calculate the total phenolic content as mg gallic acid equivalents (GAE) per 100 g.

#### 2.7.2. Total Flavonoid Content (TFC)

TFC was determined using the method described by Dewanto et al. [28], and applied as reported in reference [27]. The results were expressed in terms of catechin equivalents as mg CE per 100 g.

#### 2.7.3. Antioxidant Activities

The antioxidant capacity of the samples was determined using the CUPRAC (cupric ion reducing antioxidant capacity) method according to Apak et al. [29], with some modifications [30]. The results were expressed as mg Trolox equivalents (TE) per 100 g.

The free radical scavenging activity of the samples was determined using the DPPH (2,2-diphenyl-1-picrylhydrazyl) radical method according to Hara et al. [31]. In this assay, 150 μL of the sample was mixed with an equal volume (150 μL) of 0.1 mM DPPH solution. The mixture was vortexed briefly for approximately 10 s and then incubated in the dark at room temperature (25 °C) for 30 min. For the blank measurement, distilled water (150 μL) was used instead of the sample extract. Radical scavenging activity was calculated as percentage inhibition using the following equation:

$$\begin{array}{c} DPPH\;Radical\;Inhibition\;\left(\%\right)=\;\left(\frac{A_{Blank}-A_{Sample}}{A_{Blank}}\times 100\right) \end{array}$$

The experiment was repeated for Trolox solutions at different concentrations to prepare a calibration curve. The percentage inhibition values corresponding to Trolox equivalent (TE) were expressed as mg TE per 100 g.

### 2.8. Statistical Analyses

For the simulated digestion analysis, two independent sample bottles were prepared for each experimental condition. All analyses were performed in triplicate, and the results are expressed as mean ± standard deviation. Statistical analyses were conducted using one-way analysis of variance (ANOVA) with repeated measures, as appropriate, using SPSS software (version 28.0). Differences among samples were evaluated using Tukey’s post hoc multiple comparison test, and statistical significance was set at *p* < 0.05.

## 3. Results and Discussion

### 3.1. Bioactive Potential of PE and Inclusion Complex

Bee products, particularly pollen, are multifunctional natural sources rich in vitamins, minerals, and proteins, and they represent effective alternatives to synthetic antioxidants and antimicrobials. Their bioactive compounds, mainly phenolic acids and flavonoids, are responsible for their antioxidant, antibacterial, anti-inflammatory, and anticancer activities [32].

The total phenolic content (TPC) and total flavonoid content (TFC) of the pollen extract were determined as 2817 ± 217 mg GAE/100 g and 5255 ± 317 mg QE/100 g, respectively. These values indicate that the analyzed pollen is a rich source of phenolic and flavonoid compounds.

Comparable findings have been reported in the literature. Mutlu and Erbas [33] documented TPC values ranging from 338.15 to 2857.77 mg GAE/100 g in bee pollen samples collected from different regions of Türkiye. Notably, pollen from the Eastern Black Sea region (Bayburt province) exhibited a TPC of 2857.77 mg GAE/100 g, which closely aligns with the phenolic content observed in the present study, highlighting the high phenolic richness of pollen originating from specific geographical regions.

The antioxidant capacity of the pollen extract was evaluated using both DPPH and CUPRAC assays, revealing substantial antioxidant activity. The antioxidant capacity values were determined as 4305 ± 495 mg TE/100 g and 3685 ± 248 mg TE/100 g by the CUPRAC and DPPH methods, respectively. Differences between the two assays are expected, as each method is based on distinct reaction mechanisms and sensitivity toward specific antioxidant compounds [29,34]. Nevertheless, the consistently high values obtained by both assays clearly demonstrate the strong antioxidant potential of the pollen extract. This pronounced antioxidant activity can be attributed not only to the high total phenolic and flavonoid contents but also to the structural diversity of the bioactive compounds present in bee pollen. Recent metabolomic evidence provided by Qiao et al. [35] supports these findings, demonstrating that the antioxidant capacity of bee pollen is closely linked to its rich profile of hydroxycinnamic acid-derived phenolamides, such as *p*-coumaroyl, caffeoyl, and feruloyl polyamine conjugates, as well as a wide array of flavonoid glycosides predominantly derived from quercetin, kaempferol, isorhamnetin, and syringetin. The presence of multiple hydroxyl groups and conjugated systems within these molecules enhances their radical scavenging and redox properties, thereby contributing significantly to the overall antioxidant activity.

In addition to antioxidant properties, the antimicrobial relevance of these phenolic compounds should also be considered. Encapsulation of phenolics with BCD has been shown to enhance their antimicrobial activity by improving solubility, stability, and controlled release, thereby preserving bioactive properties against both Gram-positive and Gram-negative bacteria (e.g., protocatechuic and vanillic acid derivatives [36], gallic acid [15], phenolic extracts of bee pollen and propolis [37]). However, it is important to note that encapsulation efficiency based solely on total phenolic content may not reflect the retention of specific antimicrobial-active compounds, and thermal or digestion-related losses could reduce antimicrobial efficacy. Therefore, while the pollen extract exhibits strong antioxidant capacity, its antimicrobial performance may vary depending on the preservation of key bioactive constituents during encapsulation and digestion.

Bee pollen is known to contain more than 200 identified chemical constituents, including polyphenols and fat-soluble vitamins, which collectively influence its bioactive potential. However, the phenolic composition and antioxidant capacity of bee pollen are highly variable and depend on several factors, such as botanical origin, geographical location, climatic conditions, soil composition, and harvesting period [38]. Therefore, the variations observed between the results of this study and those reported in previous research can be reasonably attributed to differences in these environmental and botanical factors [33,39,40,41].

### 3.2. Characterization of Complexes with and Without Pollen Extract

#### 3.2.1. Zeta Potential and Particle Size Distribution

The average particle size and zeta potential values of the inclusion complexes are presented in Table 1. The results demonstrate that complex particle size increased progressively with increasing PE concentration. The mean particle size of the PE-BCD complex was 173.1 ± 1.5 nm, whereas complexes prepared at PE:BCD ratios of 1:1, 1:2, and 1:3 exhibited particle sizes of 373.5 ± 3.4 nm, 410.1 ± 5.0 nm, and 640.0 ± 2.1 nm, respectively.

The observed increase in particle size upon PE incorporation suggests that phenolic compounds interact with the BCD coating matrix through various irreversible or semi-irreversible interactions, including hydrophobic interactions, hydrogen bonding, van der Waals forces, ionic interactions, and possible covalent bonding [42]. Moreover, the ratio between the coating material and the active compound is a critical factor influencing particle size. Increasing BCD concentration likely enhances the formation of host–guest inclusion complexes and strengthens intermolecular interactions within the matrix, as previously reported for cyclodextrin-based systems [43]. Higher cyclodextrin availability shifts the complexation equilibrium toward greater inclusion efficiency, which may increase matrix density and promote particle growth. This enlargement is advantageous, as it may enhance the encapsulation efficiency and protect the bioactive compounds from aggregation or degradation during the encapsulation process [44].

The particle size of the encapsulated phenolic extracts is expected to influence their interactions with bacterial cell envelopes in addition to colloidal stability. Smaller particles provide a higher surface area and enhanced contact with bacterial membranes, which can facilitate penetration, membrane disruption, and leakage of cellular contents, ultimately improving antibacterial efficacy (e.g., nanomangosteen peel extract in chitosan [45]; Citrus reticulata peel nano-emulsions [46]). Moreover, particle size affects encapsulation efficiency and the controlled release of bioactive compounds, with smaller and well-dispersed complexes enabling more effective delivery of antimicrobial phenolics to target sites (e.g., sour cherry pomace extract [47]). Collectively, these findings suggest that the observed differences in complex size in our study (373–640 nm) may influence antibacterial activity, with smaller PE:BCD ratios potentially favoring more efficient interactions with bacterial cell envelopes.

The zeta potential values provide important insights into the surface charge characteristics and colloidal stability of the inclusion complex. Zeta potential is a critically important parameter for predicting the physicochemical stability and potential bioavailability of bioactive nanomaterials [48]. The BCD-only complex exhibited a slightly positive zeta potential (+3.66 ± 0.4 mV). In contrast, incorporation of PE led to a pronounced shift toward negative surface charge, with zeta potential values of −24.0 ± 1.3 mV, −22.0 ± 2.8 mV, and −23.5 ± 4.7 mV for PE:BCD ratios of 1:1, 1:2, and 1:3, respectively.

This shift from positive to negative zeta potential indicates that phenolic compounds present in the pollen extract significantly influence the surface properties of the BCD-based complexes. The resulting negative surface charge enhances electrostatic repulsion between particles, thereby improving colloidal stability and reducing the likelihood of aggregation. Similar trends have been reported by Hanafy et al. [42], who observed a decrease in zeta potential from −8.8 mV in empty complexes to −17.6 mV following PE loading.

The negative zeta potential can be attributed to the presence of negatively charged bioactive constituents such as flavonoids, tannins, saponins, and phenolic acids, which act as effective stabilizing agents on the complex’s surface [49]. Additionally, the negative surface charge suggests that, due to the amphiphilic structure of BCD molecules, unsubstituted hydroxyl (-OH) groups are preferentially oriented toward the aqueous environment [50]. This molecular orientation enhances surface hydrophilicity, increases water interaction capacity, and contributes to the improved dispersion stability of the nanoparticles.

The observed negative zeta potential of PE-loaded BCD complexes could influence their interactions with bacterial membranes, which are typically negatively charged due to the presence of lipopolysaccharides in Gram-negative bacteria or teichoic acids in Gram-positive bacteria [51]. Because charges repel, the negatively charged complex surfaces may reduce nonspecific electrostatic adhesion to bacterial membranes, potentially limiting direct interactions. However, other mechanisms such as hydrophobic interactions, hydrogen bonding, or specific ligand–receptor binding could still facilitate interactions. Therefore, while the negative zeta potential enhances colloidal stability, it might slightly decrease electrostatic-driven interactions with bacterial surfaces, depending on the bacterial type and the local environment [52].

#### 3.2.2. Encapsulation Efficiency

**Figure 2:**
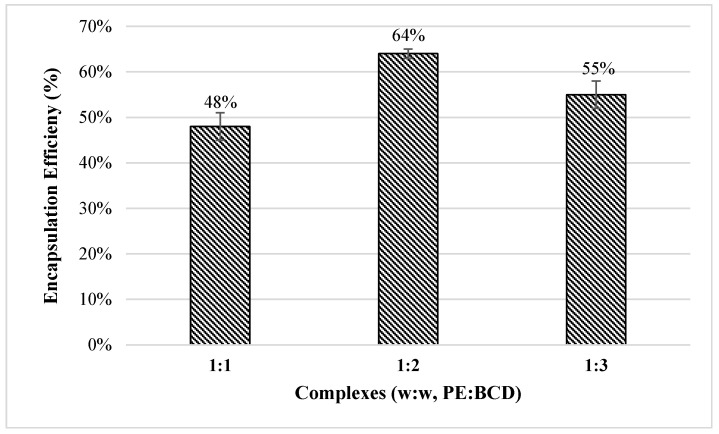
Encapsulation efficiency (%) of inclusion complexes.

The encapsulation efficiency (EE) of the complex is shown in Figure 2. Encapsulation efficiency was calculated based on the total phenolic content (TPC) of the encapsulated samples. Among the tested formulations, the complex prepared at a PE:BCD ratio of 1:2 exhibited the highest TPC value (1832 ± 30 mg GAE/100 g), which was significantly higher than those obtained for the 1:1 (1393 ± 79 mg GAE/100 g) and 1:3 (1579 ± 88 mg GAE/100 g) formulations (*p* < 0.05).

Consistent with the TPC results, the highest encapsulation efficiency was achieved at a PE: BCD ratio of 1:2, reaching 64%. In comparison, complexes prepared at PE: BCD ratios of 1:1 and 1:3 exhibited encapsulation efficiencies of 48% and 31%, respectively. Based on its superior encapsulation performance, the complex formulation with a PE:BCD ratio of 1:2 (*w*:*w*) was selected for subsequent in vitro gastrointestinal digestion and antimicrobial activity analyses.

Encapsulation efficiency of phenolic extracts is strongly dependent on the core-to-coating ratio, as both insufficient and excessive amounts of coating material can negatively affect molecular entrapment. Specifically, increasing the core compound content relative to BCD may lead to incomplete inclusion due to limited cavity availability, whereas excessive coating material may reduce the effective loading of bioactive compounds by diluting the phenolic content within the complex matrix. In this context, the lower encapsulation efficiencies observed at PE:BCD ratios of 1:1 and 1:3 in the present study can be attributed to suboptimal coating conditions.

These findings are in good agreement with the results reported by Eyüboğlu et al. [53], who demonstrated that increasing the BCD ratio significantly enhanced encapsulation efficiency only up to an optimal level (9.76% BCD), as determined by central composite design (CCD)-based optimization of *Pinus brutia* phenolic extract-BCD inclusion complexes. Beyond this optimal point, no further improvement in encapsulation efficiency was observed, underscoring the critical role of BCD concentration in achieving efficient phenolic entrapment.

Encapsulation efficiency is governed not only by the concentration of the coating material but also by the physicochemical properties of the polymer matrix and the structural characteristics of the encapsulated polyphenolic compounds [17]. Effective inclusion within the BCD cavity primarily depends on molecular size compatibility, as well as intermolecular interactions such as hydrogen bonding, van der Waals forces, and the spatial fit between the active compound and the coating material. Furthermore, the polarity of functional groups within polyphenolic molecules influences their orientation during complex formation, with nonpolar moieties preferentially accommodated within the hydrophobic cavity of BCD [54].

Comparable trends have also been reported for pollen extract encapsulation using different wall materials. Maqsoudlou et al. [44] showed that the encapsulation efficiency of pollen extracts at a 3:1 core-to-wall ratio varied significantly depending on the coating material employed, with encapsulation efficiencies of 82.51%, 78.20%, and 67.84% achieved using maltodextrin, whey protein concentrate, and their combination, respectively. In comparison, the relatively lower encapsulation efficiency observed in the present study may be attributed to differences in encapsulation technique and/or the use of BCD as the coating material, which may influence the interaction dynamics and retention efficiency of pollen-derived polyphenols within the complex matrix.

#### 3.2.3. Antimicrobial Activity

The complex formulation exhibiting the highest encapsulation efficiency, prepared at a PE:BCD ratio of 1:2 (*w*:*w*), was selected for antimicrobial analysis. The antimicrobial activity of PE, BCD-only complex, and PE-BCD complex was evaluated against *Bacillus cereus* ATCC 11778, *Escherichia coli* ATCC 25922, *Staphylococcus aureus* ATCC 25,923 and *Salmonella* Typhimurium ATCC 0402 (Table 2).

The results demonstrate that antimicrobial activity was primarily associated with PE, whereas the complex matrix alone exhibited negligible inhibitory effects (MIC and MBC > 50 mg/mL for all cultures). The obtained findings were effective when compared with certain studies using different pollen extracts; however, in other reports, lower concentrations were required to achieve the desired antimicrobial effect. For instance, among six different pollen extracts, some showed no inhibitory effect against specific microorganisms, whereas others exhibited antimicrobial activity at lower concentrations ranging from 0.31 to 2.5 mg/mL [55]. These compounds often provide multifunctional benefits and may act synergistically with other preservation strategies. Thus, even though the MIC values are relatively high in vitro, they remain relevant for practical food and nutraceutical applications.

PE showed remarkable antibacterial activity against all tested microorganisms, with MIC values ranging from 6.25 to 25 mg/mL and MBC values between 12.5 and 50 mg/mL. Among the tested strains, *B. cereus* was the most susceptible microorganism, exhibiting the lowest MIC (6.25 mg/mL) and MBC (12.5 mg/mL) values. This higher sensitivity of *B. cereus* may be attributed to the structural characteristics of Gram-positive bacteria. In contrast, *S.* Typhimurium showed the highest resistance (MIC: 25 mg/mL). This result was also consistent with the literature. The presence of an additional outer membrane in Gram-negative bacteria, composed of phospholipids, proteins, and lipopolysaccharides, acts as a selective permeability barrier that limits the penetration of most bioactive molecules, thereby resulting in the lower antimicrobial activity of pollen samples [56,57,58]. The difference in susceptibility between Gram-positive and Gram-negative bacteria is likely associated with differences in their cell wall structures. Since pollen exerts its antimicrobial activity partly through interactions with the cell wall [5,6], the structural characteristics of the microorganisms are critical. The lipopolysaccharide- and porin-rich outer membrane of Gram-negative bacteria [59,60] may reduce the ability of pollen extracts to penetrate or disrupt the cells. However, it should be emphasized that the observed differences in antimicrobial activity cannot be attributed directly to the Gram-positive or Gram-negative characteristics of the microorganisms. The antimicrobial efficacy also varies depending on the type and concentration of phenolic compounds responsible for the bioactivity [61]. Accordingly, even among microorganisms sharing the same Gram attributes, differences in susceptibility may be observed due to variations in phenolic composition and their specific modes of action.

The PE-BCD complex demonstrated antibacterial effects; however, their activity was markedly lower than that of PE. MIC values for the complex ranged between 25 and 50 mg/mL, while MBC values were consistently 50 mg/mL. Similar antibacterial activity of ethanolic pollen extracts against *S. aureus* and *E. coli* has been previously reported, supporting the antimicrobial potential of pollen-derived bioactive compounds [62,63]. The MIC values of eight different pollen samples were reported within a broad range of 1.81–33.92 mg/mL, and since antimicrobial activity is directly associated with compositional characteristics, variations depending on botanical origin, climate, and soil conditions may lead to differing results, making discrepancies with the literature expected [57].

The encapsulation of PE within the BCD matrix did not markedly enhance its antibacterial effectiveness. This reduction in activity may be related to the lower total phenolic content and antioxidant capacity of encapsulated PE compared to the free extract, as well as the controlled release behavior associated with BCD inclusion complexes. Similar decreases in antimicrobial activity following BCD encapsulation have been reported in the literature. For instance, Auezova et al. [64] observed that cyclodextrin-encapsulated phenylpropanoids (Hydroxypropyl-BCD/phenylpropanoid complexes) exhibited significantly lower antibacterial activity against *E. coli* and *Staphylococcus epidermidis* compared to their non-encapsulated counterparts. However, the underlying mechanisms responsible for this decrease have been discussed in only a limited number of studies. It has been reported that hydrophobic molecules play an important role in antimicrobial activity. Following encapsulation, strong interactions may occur between these hydrophobic fractions and the encapsulating matrix, which can reduce the release of the active molecules and consequently lead to a decrease in antimicrobial efficacy [64,65]. Eghbal et al. [66] also stated that the observed differences could be attributed to the fact that encapsulation affects the release of the core material at the desired rate, thereby influencing its biological activity.

### 3.3. In Vitro Gastrointestinal Digestion

#### 3.3.1. Phenolic Content and Antioxidant Activity

The complex formulation exhibiting the highest encapsulation efficiency, prepared at a PE:BCD ratio of 1:2 (*w*:*w*), was selected for subsequent in vitro gastrointestinal digestion experiments. The effects of in vitro simulated gastrointestinal digestion on TPC, TFC, and antioxidant capacity of the selected complex are summarized in Table 3.

A significant reduction in TPC, TFC, and antioxidant activity, as assessed by DPPH and CUPRAC assays, was observed during simulated gastrointestinal digestion of the 1:2 (*w*:*w*, PE:BCD) complex (*p* < 0.05). The TPC decreased from 1832 ± 30 mg GAE/100 g in the undigested sample to 1122 ± 25 mg GAE/100 g after the gastric phase, followed by a partial recovery to 1420 ± 23 mg GAE/100 g during the intestinal phase. In contrast, TFC and antioxidant capacity showed pronounced reductions during the gastric phase and remained relatively stable throughout the intestinal phase, suggesting a higher susceptibility of flavonoids to degradation under gastrointestinal conditions.

Given that antioxidant activity is closely associated with phenolic composition, the observed changes in DPPH and CUPRAC values likely reflect digestion-induced structural modifications and partial degradation of phenolic compounds [67]. These findings are in agreement with previous studies reporting a substantial decrease in phenolic content during gastric digestion, followed by partial recovery in the intestinal phase due to enhanced solubilization and release under near-neutral pH conditions [19,68].

The gastrointestinal stability and bioaccessibility of phenolic compounds are strongly influenced by their physicochemical properties and their interactions with the digestive environment. Acidic gastric conditions can promote the degradation or transformation of polyphenols with limited acid stability, resulting in decreased TPC, TFC, and antioxidant activity. During the intestinal phase, the transition to neutral pH, combined with the presence of pancreatic enzymes and bile salts, facilitates the release, solubilization, and partial recovery of phenolic compounds [69]. In contrast, free phenolic compounds, such as those present in unencapsulated pollen, generally exhibit low bioaccessibility. For example, Aylanc et al. [68] reported that following in vitro digestion, only approximately 31% of TPC and 25% of TFC from pollen became bioaccessible.

Encapsulation with BCD provides a protective effect by forming inclusion complexes with phenolic compounds, thereby shielding them from acidic degradation in the gastric phase and enhancing their solubility and stability during intestinal digestion. This encapsulation strategy enables controlled release of phenolics and improves their bioaccessibility and potential bioavailability. Supporting this mechanism, Hızır-Kadı et al. [70] demonstrated that liposomal encapsulation of pollen phenolic extracts increased TPC and TFC bioaccessibility by approximately two- and four-fold, respectively. In the present study, the bioaccessibility of TPC, TFC, DPPH, and CUPRAC was determined as 81%, 33%, 48%, and 76%, respectively, indicating a marked improvement compared to values reported for unencapsulated pollen in the literature [68,71]. This enhancement can be attributed to the protective and solubilizing effects of BCD, which facilitate the stabilization and release of bioactive compounds during gastrointestinal digestion.

#### 3.3.2. Determination and Identification of Phenolic Compounds

The phenolic compound profile of the encapsulated PE and the effect of simulated in vitro gastrointestinal digestion on individual phenolic compounds are presented in Table 4. Six phenolic compounds were identified in the PE-BCD inclusion complex, namely trans-cinnamic acid, chlorogenic acid, *p*-coumaric acid, caffeic acid phenethyl ester (CAPE), quercetin-3-O-glucoside, and quercetin.

The identified phenolic profile is consistent with previous reports on bee pollen composition. Qiao et al. [35] identified 25 flavonoid glycosides in bee pollen samples, with quercetin, kaempferol, isorhamnetin, and syringetin as the predominant aglycones. Similarly, Hanafy, Salim et al. [72] reported that bee pollen extract contained the highest level of quercetin (6.43 μg/mL) and lower amounts of vanillin (0.082 μg/mL), along with other phenolics including cinnamic acid (3.89), syringic acid (3.84), catechin (1.42), gallic acid (1.38), taxifolin (0.7), ferulic acid (0.39), pyrocatechol (0.32), naringenin (0.31), *p*-coumaric acid (0.27), rutin (0.26), chlorogenic acid (0.24), methyl gallate (0.12), and caffeic acid (0.09). Similarly, Mutlu & Erbas [33] further identified a broad range of phenolic acids and flavonoids, including *t*-cinnamic acid, *p*-coumaric acid, rutin, quercetin, luteolin, kaempferol, and caffeic acid. Comparison of these findings with the present results suggests that encapsulation and processing may influence the detectable concentration and stability of individual phenolic compounds.

Before digestion, CAPE was the predominant phenolic acid in the encapsulated extract, with a concentration of 355 ± 2.5 mg/100 g. Among flavonoids, quercetin-3-O-glucoside (235.5 ± 4.9 mg/100 g) and quercetin (100.8 ± 6.4 mg/100 g) were the most abundant compounds. During simulated gastrointestinal digestion, several phenolic compounds, including *t*-cinnamic acid, *p*-coumaric acid, and CAPE, were no longer detectable after the gastric phase, indicating their susceptibility to acidic conditions and enzymatic degradation.

In contrast, chlorogenic acid, quercetin-3-O-glucoside, and quercetin showed comparatively higher stability during digestion. Chlorogenic acid content decreased markedly during the gastric phase (from 12.0 to 2.2 mg/100 g) but partially recovered in the intestinal phase (6.2 mg/100 g), suggesting enhanced solubilization or release under intestinal conditions. No statistically significant changes were observed in the concentrations of quercetin-3-O-glucoside and quercetin during the gastric phase; however, both compounds exhibited a gradual decline during the intestinal phase.

The differential stability of phenolic acids and flavonoids during digestion can be attributed to variations in their chemical structures, including molecular size, degree of conjugation, glycosylation, and susceptibility to pH- and enzyme-induced transformations [68,73]. Glycosylated flavonoids such as quercetin-3-O-glucoside generally display greater stability under gastric conditions, whereas aglycones and esterified phenolics, such as CAPE, are more prone to degradation. The higher stability of quercetin and its derivatives (e.g., quercetin-3-O-glucoside) during in vitro gastrointestinal digestion can be attributed to their molecular structure and conjugation with sugar moieties, which partially protect the flavonoid core from acidic and enzymatic degradation [74]. The higher stability of quercetin and its derivatives (e.g., quercetin-3-O-glucoside) during in vitro gastrointestinal digestion is linked not only to their antioxidant behavior but also to their broad-spectrum antimicrobial potential. Quercetin exhibits inhibitory effects on diverse microorganisms, such as Gram-positive and Gram-negative bacteria, as well as fungi and viruses [75], while other phenolic acids such as caffeic acid, gallic acid, and chlorogenic acid also exhibit antimicrobial properties, though their effectiveness is more variable [76]. The preserved structure of quercetin derivatives during digestion may thus allow for sustained interaction with microbial targets, potentially maintaining their bioactive function in the gastrointestinal environment.

In contrast, simple phenolic acids such as *t*-cinnamic acid, *p*-coumaric acid, and the ester CAPE are more prone to hydrolysis under gastric conditions, with CAPE being fully degraded due to its ester linkage [73]. Additionally, the partial hydrophilicity and aromatic structure of quercetin derivatives favor inclusion within the hydrophobic cavity of BCD [77], providing further protection, whereas smaller, more polar phenolics interact less effectively with BCD and are rapidly lost during gastric digestion.

Overall, the observed changes in individual phenolic compounds during digestion are in good agreement with the trends reported for total phenolic and flavonoid contents (Table 4), where a partial recovery of TPC and a sustained decrease in TFC were observed from the gastric to the intestinal phase. These findings highlight the selective protective effect of BCD encapsulation on specific phenolic compounds and underscore the importance of compound-specific behavior in determining the bioaccessibility of phenolics during gastrointestinal digestion.

## 4. Conclusions

In this study, PE obtained from the Eastern Anatolia region of Türkiye was demonstrated to be a rich source of phenolic compounds and flavonoids, exhibiting strong antioxidant and moderate antimicrobial activity. Encapsulation of the pollen extract within a BCD matrix significantly influenced its physicochemical properties, encapsulation efficiency, biological activity, and gastrointestinal stability. Among the tested formulations, the PE:BCD ratio of 1:2 (*w*:*w*) was identified as the optimal formulation, providing the highest encapsulation efficiency, improved colloidal stability through enhanced negative surface charge, and effective protection of bioactive compounds. Although encapsulation slightly reduced the immediate antimicrobial activity compared to the free extract, it markedly improved the gastrointestinal stability and bioaccessibility of phenolic compounds, particularly total phenolics and antioxidant capacity. The in vitro digestion results revealed that BCD encapsulation mitigated degradation under gastric conditions and facilitated partial recovery and controlled release of phenolics during the intestinal phase, leading to substantially higher bioaccessibility values than those reported for unencapsulated pollen. Furthermore, compound-specific analysis showed that BCD selectively protected structurally stable phenolics, such as chlorogenic acid and quercetin derivatives, highlighting the importance of molecular structure in digestion behavior. Thus, while BCD-based encapsulation enhances the bioaccessibility and stability of pollen-derived bioactives, it may compromise their immediate antibacterial efficacy, representing a trade-off between protection and rapid antimicrobial action. However, a significant limitation of the present study is the absence of post-digestion antimicrobial assessment, which restricts the microbiological interpretation of the functional performance of encapsulated extracts after gastrointestinal simulation. Future studies should therefore evaluate antimicrobial activity following in vitro digestion to better elucidate the biological relevance of the improved bioaccessibility observed. Overall, these findings indicate that β-cyclodextrin-based encapsulation is an effective strategy to enhance the functional performance of pollen-derived bioactives, supporting their potential application as natural antioxidant ingredients in functional foods, nutraceuticals, and pharmaceutical formulations.

## Figures and Tables

**Table 1:** Particle size and zeta potential of BCD complexes prepared with different PE:BCD ratios (*w*:*w*).

	BCD Complex	PE-BCD Complexes (*w*:*w*, PE:BCD)		
		1:1	1:2	1:3
Particle Size (nm)	173.1 ± 1.5 ^d^	373.5 ± 3.4 ^c^	410.1 ± 5.0 ^b^	640.0 ± 2.1 ^a^
Zeta Potential (mV)	3.66 ± 0.4 ^a^	−24.0 ± 1.3 ^c^	−22.0 ± 2.8 ^b^	−23.5 ± 4.7 ^c^

PE: pollen extract; BCD: β-cyclodextrin. Data are presented as mean ± standard deviation (*n* = 3). ^a–c^ Lowercase letters within a row indicate statistically significant differences between groups (*p* < 0.05).

**Table 2:** Minimum inhibitory concentration (MIC) and minimum bactericidal concentration (MBC) (mg/mL) of pollen extract (PE), BCD complex, and PE-BCD complex against selected bacterial strains.

Microorganisms	PE		BCD Complex		PE-BCD Complex	
	MIC	MBC	MIC	MBC	MIC	MBC
*Bacillus cereus* ATCC 11778	6.25	12.5	>50	>50	50	50
*Escherichia coli* ATCC 25922	12.5	50	>50	>50	25	50
*Staphylococcus aureus* ATCC 25923	12.5	50	>50	>50	25	50
*Salmonella* Typhimurium ATCC 0402	25	50	>50	>50	50	50

“>50” indicates no inhibitory or bactericidal effect at the highest tested concentration.

**Table 3:** TPC and antioxidant activity during in vitro gastrointestinal digestion of 1:2 (*w*:*w*, PE:BCD) complex.

	Initial	Gastric Phase	Intestinal Phase	Bioaccessibility (%)
TPC (mg GAE/100 g)	1832 ± 30 ^a^	1122 ± 25 ^c^	1420 ± 23 ^b^	81
TFC (mg QE/100 g)	2146 ± 56 ^a^	719 ± 60 ^b^	716 ± 28 ^b^	33
DPPH (mg TE/100 g)	3600 ± 257 ^a^	1817 ± 246 ^b^	1719 ± 88 ^b^	48
CUPRAC (mg TE/100 g)	3500 ± 121 ^a^	2560 ± 82 ^b^	2650 ± 120 ^b^	76

Values are expressed as mean ± SD of three analytical replicates from two independent complex batches. Data are presented as mean ± standard deviation (n = 3). ^a–c^ Lowercase letters within a row indicate statistically significant differences between groups (*p* < 0.05).

**Table 4:** The effect of in vitro digestion on individual phenolic compounds of the 1:2 (*w*:*w*, PE:BCD) complex.

Phenolic Compound	Initial	Gastric Phase	Intestinal Phase
*t*-Cinnamic acid	43.3 ± 0.7 ^a^	–	–
Chlorogenic acid	12 ± 0.8 ^a^	2.2 ± 0.2 ^c^	6.2 ± 0.9 ^b^
*p*-Coumaric acid	13.3 ± 1.6 ^a^	–	–
Caffeic Acid Phenethyl Ester (CAPE)	355 ± 2.5 ^a^	–	–
Quercetin-3-O-glucoside	235.5 ± 4.9 ^a^	93.0 ± 0.5 ^a^	65.5 ± 0.4 ^b^
Quercetin	100.8 ± 6.4 ^a^	72.5 ± 7.8 ^a^	50.7 ± 1 ^b^

Values are expressed as mean ± SD of three analytical replicates from two independent complex batches. Data are presented as mean ± standard deviation (n = 3). ^a–c^ Lowercase letters within a row indicate statistically significant differences between groups (*p* < 0.05).

## Data Availability

The original contributions presented in the study are included in the article, further inquiries can be directed to the corresponding authors.
